# From bureaucracy to bedside teamwork: maintaining patient safety in COVID-19 care - staff experiences of COVID-19 care in a Swedish university hospital

**DOI:** 10.1186/s12913-025-13555-2

**Published:** 2025-10-21

**Authors:** Kristina Rosengren

**Affiliations:** 1 Department of Internal Medicine Mölndal, Sahlgrenska University Hospital, Gothenburg, SE-413 45 Sweden; 2 Institute of Health and Care Sciences, Sahlgrenska Academy, University of Gothenburg, Gothenburg, SE-405 30 Sweden

**Keywords:** COVID-19, Staff, Teamwork, Qualitative content analysis, University hospital, Working environment

## Abstract

**Purpose:**

This study aimed to describe healthcare staff’s experiences of planning and providing care for patients with COVID-19 at a university hospital in western Sweden.

**Design:**

A qualitative content analysis was conducted based on 28 semi-structured interviews with healthcare staff at a university hospital in western Sweden.

**Findings:**

One overarching category was identified: *Togetherness to Maintain Patient Safety*, comprising three subcategories: *Managing Uncertainty and Fear of an Unknown Disease*, *Coping with High Workloads in an Unfamiliar Work Environment*, and *Collaborating with unfamiliar staff members*. These themes illustrate how staff navigated the challenges of planning and delivering care during the pandemic.

**Originality:**

Despite severe challenges, including high patient acuity, limited evidence-based knowledge, and resource constraints, staff successfully implemented ongoing improvements through a shared commitment to patient safety. Organizational flexibility and the reduction of hierarchical routines alleviated burdens on healthcare professionals, fostering collaboration and a person-centred approach to care.

**Research implications:**

Collaboration among staff emerged as a central feature, supported by reduced bureaucracy and the use of continuous improvement methods, such as the plan–do–study–act cycle.

**Practical implications:**

The study highlights the challenges created by the rapid onset of the COVID-19 pandemic, which profoundly affected healthcare working conditions. Staff collaboration, reinforced by less bureaucratic structures, facilitated continuous improvement and strengthened the commitment to delivering safe care under uncertain and demanding circumstances.

**Social implications:**

Working together, supported by effective organizational and managerial leadership, was essential for adapting practices and safeguarding patient safety when evidence-based guidelines were limited. Standardized group introductions, divided into general routines and unit-specific practices, were identified as critical for reducing workload pressures on both permanent and redeployed staff.

## Background

The COVID-19 pandemic placed a substantial burden on healthcare systems globally, driven by interrelated challenges including a high influx of critically ill patients, limited evidence-based knowledge, and constrained resources. This study contributes to the literature on staffing, care planning, and clinical practice during rapidly evolving and unprecedented healthcare crises such as the COVID-19 pandemic.

The emergence of the novel coronavirus SARS-CoV-2 (COVID-19) presented an extraordinary challenge to health systems worldwide. The disease manifests across a wide clinical spectrum, from asymptomatic infection to severe illness and death. Healthcare services were placed under intense pressure due to surges in critically ill patients requiring intensive care and the lack of conclusive evidence on effective treatment strategies [1]. In response, preventive measures such as hand hygiene, antiseptic protocols, and the use of personal protective equipment (PPE) were widely implemented, alongside medical interventions targeting respiratory and circulatory complications [2, 3]. This study seeks to deepen understanding of how the rapid onset of the pandemic affected healthcare professionals’ working conditions.

A pandemic is defined as the global spread of a novel disease affecting a substantial proportion of the population [4]. Historical examples—including the Spanish flu (1918–1921), the Asian flu (1957), the Hong Kong flu (1968), and the SARS outbreak (2003) underscore the recurring and transboundary nature of pandemics [5]. Research on previous pandemics, such as SARS, highlights the critical importance of managerial actions in ensuring access to PPE and providing clear antiseptic guidelines [6]. COVID-19, first identified in late 2019 in Wuhan, China, was declared a global pandemic by March 2020 [3, 4]. The disease led to severe acute respiratory syndrome and, in the most critical cases, death [3]. Professional nursing played a vital role in the care of seriously ill patients, often under new and highly challenging working conditions [7]. While healthcare systems ordinarily rely on structured routines, the sudden emergence of COVID-19 underscored the importance of adaptive teamwork and managerial competence in safeguarding patient safety [8–10]. Limited PPE availability during the early phases of the pandemic heightened health professionals’ concerns about infection risk [11]. Working conditions were further strained by rapidly evolving information and guidelines, resulting in heavier workloads, time-intensive planning, and insufficient recovery time [12]. Numerous studies reported significant mental health challenges among healthcare workers in this period [13–15]. For example, Iranian nurses reported elevated levels of mental, physical, and temporal stress, as well as frustration, compared with other healthcare professionals [14]. Mental health impacts such as depression, anxiety, insomnia, and moral distress were widely documented [16–20]. In the United States, physical activity and exercise were adopted as coping strategies to manage psychological distress [21]. At the organizational level, the pandemic produced both negative outcomes, heightened demands and resource scarcity and positive aspects, including an enhanced sense of purpose and professional meaning [22]. Psychosocial support and adequate leisure time were identified as essential for helping healthcare professionals manage sustained demands. Prioritizing mental health for social workers and other healthcare staff during periods of crisis and instability was also emphasized [23].

In long-term care facilities, insufficient PPE supplies further increased the risk of SARS-CoV-2 infection among healthcare workers [24]. Occupational safety emerged as a critical concern, particularly in crowded transportation settings and inadequately protected workplaces. The implementation of COVID-19 prevention measures, strategic scheduling, workplace adaptations to avoid crowding, and strict adherence to hygiene protocols, was regarded as essential for mitigating morbidity and mortality and supporting economic and societal recovery [25]. In Sweden, research [26] has shown that healthcare professionals were concerned about becoming infected and subsequently transmitting the virus to others. These job demands, emotional and cognitive, combined with increased workloads, were primarily managed through collegial support [27]. Against this backdrop, the present study aimed to describe healthcare staff’s experiences of planning and providing care for patients with COVID-19 at a university hospital in western Sweden.

## Methods

### Setting

The study was conducted at a university hospital in western Sweden employing approximately 17,000 staff members across 120 departments and operating on four sites. During the pandemic, patients diagnosed with COVID-19 entered through the emergency department (ED) and were assigned to appropriate care pathways—intensive care units (ICUs) or designated COVID-19 wards, based on illness severity.

### Design

A qualitative content analysis was employed to explore and interpret healthcare staff’s experiences, perspectives, and reflections as they planned and provided care for patients with COVID-19 [28, 29]. This approach was chosen to capture the nuanced, subjective dimensions of the participants’ work environment during the pandemic [30]. The methodology emphasizes interpretation, meaning-making, and context, supporting a deep understanding of complex phenomena such as COVID-19. To ensure rigor, the study adhered to principles of trustworthiness, including credibility, dependability, and transferability. An inductive analytical approach guided interpretation, allowing themes and categories to emerge from participants’ narratives [30].

### Data collection

A strategic sampling strategy appropriate for qualitative research was used to recruit participants [30]. Inclusion criteria were: age ≥ 18 years; employment as a healthcare professional, administrator, or manager at the university hospital; at least four months of experience working during the COVID-19 pandemic; and fluency in Swedish. Staff without direct COVID-19 care experience and those employed at other hospitals were excluded. Recruitment was conducted via email, with one reminder. All respondents who met eligibility criteria and expressed interest (*n* = 28) were included. Informed consent was obtained in accordance with the General Data Protection Regulation (GDPR) [31], following provision of detailed information about the study’s purpose, methods, and intended use of findings.

According to national regulations [27, 28, 32, 33], formal ethical approval was not required because participants were engaged in routine quality-improvement activities (e.g., iterative adjustments to staffing, treatment, and care practices). These activities are often based on practice and trial-and-error due to limited evidence at the onset of the pandemic aimed at enhancing patient safety and did not involve collection of sensitive personal data. Semi-structured interviews were conducted in Swedish between May and August 2020. The interview guide included eight main questions. First, participants were asked about their background (age, education, professional experience). Second, an open-ended question was posed: “If I say COVID-19, what are your experiences from working at the hospital?” Follow-up questions were tailored to each participant’s responses. The interviews further explored (3) organizational preparedness, (4) teamwork, (5) learning experiences, and (6) strategies for managing rapid changes in the future and concluded with (7) an invitation to share additional COVID-19 experiences not previously addressed. All interviews were audio-recorded and transcribed verbatim.

The sample comprised assistant nurses (*n* = 5, all female), registered nurses (*n* = 6; five female, one male), physicians (*n* = 5; two female, three male), physiotherapists (*n* = 2, both female), micro- and meso-level managers (*n* = 5; three female, two male), and administrators (*n* = 5; two female, three male). In total, 28 participants were included, representing the emergency department (*n* = 6), wards (*n* = 11), intensive care units (*n* = 8), and human resources (*n* = 3). Participants ranged in age from 21 to 65 years (median = 49) and had between 1 and 43 years of healthcare experience (median = 21).

### Data analysis

Transcribed interviews were analyzed using qualitative content analysis as described by Graneheim and Lundman [28, 29]. The process was carried out by a senior researcher (PhD, Associate Professor) with expertise in healthcare (registered nurse) and in healthcare organization, leadership, and quality improvement. Analysis began with repeated readings of the transcripts to achieve an overall understanding of the data, particularly concerning the planning and delivery of COVID-19 care. Meaning units relevant to the study aim were identified inductively, condensed, and assigned descriptive codes, yielding 69 codes. Codes were then grouped by similarity and pattern to develop broader thematic categories. Through this iterative and interpretive process, one main category—*Togetherness to Maintain Patient Safety*—was identified, supported by three subcategories: *Managing Uncertainty and Fear of an Unknown Disease*, *Managing High Workload within an Unfamiliar Working Situation*, and *Collaborating with Unfamiliar Staff Members*. These categories captured core aspects of staff experiences during the pandemic and were illustrated with representative quotations (Table 1).

**Table 1:** Examples of data analysis

Meaning unit	Condensation	Code	Subcategory	Category
You must dare…that we really believe in these people. These patients are extremely sick…we didn’t know that at the beginning…had a meeting with nurses who knew a lot about the patient group, there was a doctor who knew a little and there was someone from rehabilitation and so you had to act based on the skills…we don’t usually do it like that…this becomes more that we can talk about this, we get together, I think it’s absolutely fantastic that it worked.	Must dare…really believe in these people, patients extremely sick, didn’t know at the beginning, meeting with nurses knew patient group, physician knew little, rehabilitation, act on skills…don’t usually do, talk, together, fantastic worked.	Uncertainty	Managing uncertainty and fear of an unknown disease	Togetherness to maintain patient safety
The manager just called and said that tomorrow it will be like that…a disaster going on and then there are rapid changes and you have to adapt…all the patients have to be handled, we can’t say no…we have to have more understanding when you work in healthcare…you have to receive patients very quickly…mentally prepare as much as you can and work on it, right?	Manager called, tomorrow, disaster, rapid changes, must adapt…all patients handled, can’t say no…more understanding work healthcare, receive patients very quickly, mentally prepare as you can.	Workload	Managing high workload within an unfamiliar working situation	Togetherness to maintain patient safety
More cooperation and more communication, you learn after 1–2 days how to communicate, we wrote notes, talked to each other, how do you feel, do you want to get out (isolated work zone), caring for each other… if you would like to go out, I can be here…go to the toilet or are drink something or having difficulty breathing (protective clothing, face mask)	Cooperation, learn to communicate, wrote notes, caring for each other, talked, how do you feel, toilet, drink, difficult breathing (protective clothing, face mask)	Communicate	Collaborating with unfamiliar staff members	Togetherness to maintain patient safety

### Human ethics and consent to participate declarations

Human ethics and consent to participate declarations are not fully applicable in this study. Ethics approval was deemed unnecessary according to national regulations [32, 33]. Formal ethical approval by an institutional review board (IRB) are not required as it was conducted within the framework of quality improvement without the collection of sensitive personal health data. Approval of the study was granted by the hospital’s board, and additional permission was obtained from the managers of the participating departments. Moreover, the study was conducted in compliance with the Helsinki Declaration [34]. Written informed consent was obtained from all participants following the provision of detailed information regarding the study’s aim, methodology, and intended use of the results.

Although IRB approval was not mandatory, ethical principles were rigorously upheld throughout the study. Emphasis was placed on respecting the rights and well-being of participating staff members, in alignment with GDPR [31]. To safeguard participant confidentiality and ensure integrity and autonomy, all participation was entirely voluntary. The data has been anonymized, and the findings are presented at the group level. No personal identifiers are included in the reporting of quotations or results, thereby minimizing the risk of harm and ensuring the protection of participants’ privacy and dignity [31, 34].

## Results

**Fig. 1:**
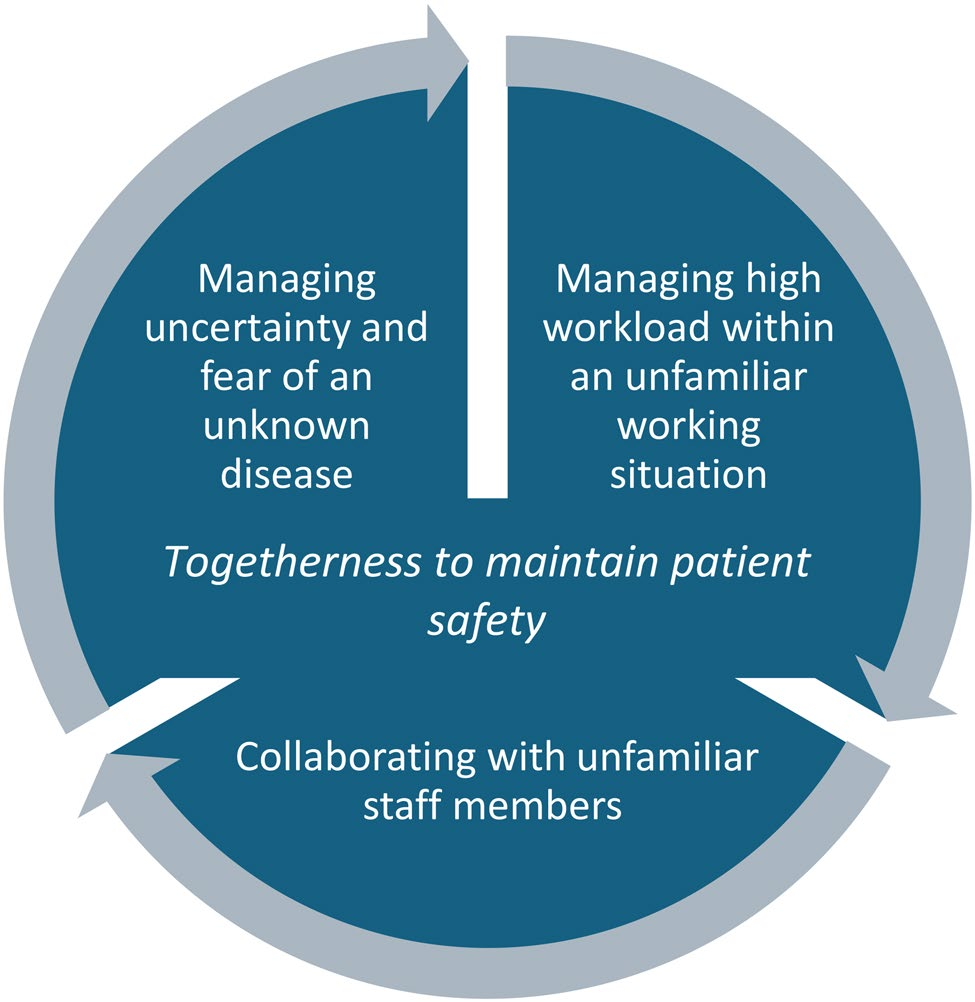
One category and three sub-categories generated in the results

The results are presented in accordance with the main category, *Togetherness to maintain patient safety* (Fig. 1), and three subcategories: *Managing uncertainty and fear of an unknown disease, Managing high workload within an unfamiliar working situation*, and *Collaborating with unfamiliar staff members.* These themes offer insights into how staff planned and delivered care to patients with COVID-19.

### Togetherness to maintain patient safety

The overarching category, *Togetherness to maintain patient safety*, emerged as a unifying perspective within the university hospital, grounded in collaboration among all stakeholders and aligned with continuous quality improvement principles such as the plan–do–study–act (PDSA) cycle. The limited availability of evidence and treatment options for severely ill patients with COVID-19 necessitated ongoing adaptations to compensate for knowledge gaps. These adaptations included the continuous introduction of new routines and the redeployment of staff from wards and outpatient clinics to unfamiliar environments, such as COVID-19 wards and intensive care units (ICUs). These challenges fostered a strong sense of solidarity, with staff emphasizing collaboration over hierarchy to manage strained working conditions, including shortages of both medication and protective equipment. No one was an expert in the novel situation, which led to collective efforts to manage high workloads and ensure patient safety.

### Managing uncertainty and fear of an unknown disease

This subcategory, *managing uncertainty and fear of an unknown disease*, captures the fear and confusion provoked by the rapid global spread of COVID-19. Staff members were notified abruptly of the evolving disaster, requiring immediate adaptation and full engagement in patient care without refusal. Participants described the lack of evidence-based guidelines, rapidly changing routines, short-term planning, and absence of centralized communication channels as major stressors. Uncertainty in the workplace was mirrored in participants’ personal lives, where anxiety extended beyond the clinical setting and negatively affected their mental health. Consequently, participants emphasized the need for recovery and opportunities to “catch their breath” in order to cope with the strained working environment and to maintain patient safety. Initially, some participants underestimated the severity of the disease. However, as cases increased and severely ill patients filled the wards, their fear intensified. One registered nurse described the situation as follows:

This virus is ruthless and like Russian roulette. It is like an addiction; it does not matter if you do not belong to a risk group; you might end up in ICU.

Health professionals also noted the significant physical decline of COVID-19 patients, requiring prolonged hospitalization and intensive care:

They are sick and incredibly weak, requiring 30 to over 50 days of care… It happens quite quickly that they become so weak. And so tired, one of the symptoms of COVID is such great fatigue… when you try to mobilize them, they are in a miserable state. (Physiotherapist)

Workplace conditions were exacerbated by the absence of clear infection control routines and patients’ reluctance to disclose flu-like symptoms, particularly in the emergency department. Organizational challenges such as unclear decision-making structures and fluctuating protective equipment guidelines contributed to virus transmission and increased workload. Therefore, participants stressed the need for clear guidelines and protocols to manage specific issues, rather than relying on constant trial and error in care activities and frequently changing routines. Moreover, they expressed frustration with the additional administrative burden, which interfered with their core clinical responsibilities.

…It is a sick person who needs to get out of bed… It is just this process that you cannot break because of COVID, which is new but not really anything different… Working with so much protective clothing, which is hard, and no relatives allowed to visit… we sign our names on the protective clothing so that they can look at our name… it feels like they feel safe with us. (Assistant Nurse)

The absence of dependable evidence compelled staff to rely on diverse information sources such as personal networks, social media, email, and websites. Despite the inconsistency of information, participants reported gradual improvements in their knowledge and critical thinking skills through trial and error:

…A lot of medical app… yes, all kinds of equipment and sounds all the time…a lot to keep in your head. We usually follow nutrition, but not like this… trach and arterial needle without experience, you do not know what you are looking for and when. (Registered Nurse)

Participants expressed disappointment over the public’s failure to adhere to social distancing guidelines, which increased the burden on healthcare services. Many participants altered their transportation and living arrangements to reduce the risk of infection, a response influenced by the absence of vaccination as well as the lack of clear instructions and guidelines from national authorities such as the Public Health Agency and the National Board of Health and Welfare. Across narratives, clear communication, reliable information, and organizational consistency were identified as critical for mitigating fear and uncertainty.

### Managing high workload within an unfamiliar working situation

The subcategory *“Managing high workload within an unfamiliar working situation”* highlights the intensification of demands resulting from the use of PPE, increased responsibilities, and the high volume of severely ill patients, all of which contributed to a strained working environment. One nurse recounted that her recovery took place at home:

I do not know what I would have done if I had not had my husband who cooked, cleaned, arranged everything, because I cannot bear anything… I want to be a nice person, I love my husband, but I get angry, I cry, I become completely unaccountable, just because you are so tired all the time… You manage to sharpen yourself because you must do a respectable job. I am a good ICU nurse. I must save patients’ lives. (Specialized ICU-nurse)

The limited supply of PPE forced staff in high-risk zones (“dirty zones”) to manage all patient care tasks without assistance from colleagues in lower-risk zones (“clean zones”). Inconsistent PPE guidelines, varied equipment brands, and uncomfortable protective gear led to heat exhaustion, skin abrasions, fogged glasses, headaches, and communication difficulties. These barriers necessitated meticulous planning for basic needs such as hydration, eating, and restroom use:

…A limit of 2 hours because you get foggy, you cannot make decisions… You are incredibly warm, you do not hear anything… just like being in a jar… You talk in short commands… do not have time for light niceness talk, use clear articulation to hear something. (Registered Nurse)

Staffing shortages due to the influx of critically ill patients resulted in the formation of a resource pool managed jointly by first-line managers and the human resources department. However, participants emphasized the limitations of staff planning processes when personnel were reallocated from elective care to support overwhelmed wards:

…There has never been anything that affected us like this pandemic… We put all aside… worked spontaneously… just had to solve what comes up… everyone worked around the clock… 48-hour week… split into two twelve-hour shifts per day, it was incredibly heavy. (Manager)

Specialist nurses and physicians experienced a dual burden: managing more patients than usual while simultaneously supervising redeployed colleagues with limited experience. This led to considerable ethical and psychological stress:

…You are the only ICU nurse; it is like crazy. Everyone asks everything all the time… It is a huge strain… You lose completely, become completely apathetic… you alternate between laughing at strange things and crying over each other because you are completely exhausted… We had 18 patients (5 ordinary beds), it was too much all the time. (Specialized ICU-nurse).

Elective and planned care activities, including outpatient services and research, were postponed, and staff were reassigned to frontline COVID-19 care:

=I was doing research but had to cancel and schedule medical staff to COVID units and emergency department. I only work with COVID, no ordinary work at all. (Physician).

### Collaborating with unfamiliar staff members

This sub-category, *collaborating with unfamiliar staff members*, describes the challenges and opportunities associated with collaboration among staff members redeployed from various departments, elective care. The transition required rapid adaptation to new colleagues, unfamiliar environments, and differing routines. However, collaboration across different hospital stakeholders posed challenges for both regular COVID-19 unit staff and relocated personnel. For example, repetitive introductions were described as an additional burden within an already strained working environment. Despite these initial disruptions, a collaborative spirit gradually emerged, characterized by mutual support, shared learning, and task redistribution. Managers played a key role in coordinating staff scheduling and integrating part-time and full-time personnel effectively:

…Who does what and how does we get routines implemented? And there was staff turnover… Communication with different departments was significant, how to distribute staff on schedule in an effective way… We got more staff to use to. (Manager)

Hierarchical boundaries diminished as healthcare professionals, administrators, and managers worked together in shared roles, focusing on patient safety. Participants described a shift from bureaucratic processes to a more fluid, team-based model. Traditional structures were temporarily dismantled to enable swift decision-making and action:

…We have a learning climate, no one is a ‘besserwisser’ or a bully who sees himself as a hero… it is teamwork… criticism is constructive, it is forward. (Physician)

Staff members, regardless of their professional background, engaged in both curative and supportive care. Examples included orthopedic surgeons, administrative personnel, and hospital managers working together in non-traditional roles. This sense of unity, a collective “us”, was seen as a return to the hospital’s foundational values, prior to the adoption of new public management principles. The pandemic, despite its challenges, facilitated renewed collaboration and strengthened interpersonal bonds:

…We really get along. You feel a certain togetherness because you work with this, it applies to all categories… No one is shying away from working… It is a management issue to create a climate and atmosphere… The summer will be tough for all of us… But better times are coming. (Physician)

Participants emphasized the need for recovery, both for patients and for themselves. They stressed the importance of careful organization and forward planning to improve the working environment, highlighting the role of managerial leadership at the micro-, meso-, and macro-levels in maintaining job satisfaction, teamwork, and collaborative healthcare practices. This was considered particularly significant by the participants given that the focus on COVID-19 care led to extended waiting lists for other healthcare services, such as hip replacements, as well as delays in research activities aimed at strengthening evidence-based practice.

## Discussion

This study successfully achieved its aim by describing healthcare staff’s experiences in planning and caring for patients with COVID-19 in Sweden. The results highlight the necessity of *togetherness* to maintain patient safety, fostering a unique “us” perspective that emerged from the limited knowledge of COVID-19. This lack of expertise compelled all staff to collaborate and mobilize every available resource. Health professionals and administrators worked in close cooperation to address the uncertainties and fears experienced by staff, patients, and their relatives.

The high number of severely ill patients suffering from an unknown viral disease created a strained working environment. Challenges such as limited protective equipment and treatment options, exacerbated by a lack of evidence, established routines, and available medications, intensified the need for collaboration across all hospital stakeholders. Staff were relocated to ICU, emergency departments, and COVID-19 wards, with around-the-clock schedules introduced to manage the influx of critically ill patients. This relocation added complexity, including unfamiliar settings, routines, and workflows, and required supervision by permanent staff, in accordance with findings from other studies [26, 27, 35, 36]. The result was a double burden and an increased workload for both relocated and existing staff, compounded by the onboarding of new colleagues and continued uncertainty due to the lack of evidence-based knowledge. The study addressed the challenges related to managing staff uncertainty and fear when dealing with a new, unknown virus [4]. Health professionals, typically guided by evidence-based practices rooted in research and clinical guidelines, were forced to abandon traditional approaches. With no established protocols or experts to consult, professionals had to adapt rapidly. Despite these limitations, their education and training enabled them to care and cure effectively [9, 11]. The lack of knowledge about COVID-19 underscores the need to improve information and support systems to reduce uncertainty and fear during future pandemics.

Insights from previous pandemics, such as the Spanish flu, Asian flu, Hong Kong flu, and SARS, should be integrated into future preparedness strategies [5, 6]. Recovery, including time away from work, is essential to adapt to new realities and maintain long-term resilience [37, 38]. Research [1, 27, 36, 39] emphasizes that collegial support and recognition of meaningful work, together with opportunities for vacation and rest, are vital to sustaining healthcare staff’s commitment and mental health during rapidly evolving crises. A robust organizational structure [11, 22, 38–40] is necessary, one that enables critical dialogue around risks and preparedness, supports clear communication, and ensures rapid deployment of reliable information channels. Establishing protocols for answering the questions WHO, HOW, WHEN, and WHY at the care unit level provides a foundation for effective pandemic response planning. Uniform, timely communication across organizational levels (micro, meso, macro) is essential for maintaining patient safety. For example, shift-specific information, delivered by team leaders who directly coordinate with senior management, ensures consistency across departments. Research [41, 42] highlights the importance of digital communication tools in decentralized healthcare systems to ensure efficiency, accessibility, and patient safety.

However, reforms are needed to optimize resource use and improve health outcomes in both the short and long term through effective vertical policy coordination [10, 34, 35, 38, 43, 44]. Crisis management strategies [40] may serve as a valuable tool for hospital managers, enabling them to act with courage, support, and sensitivity when reorganizing and transforming healthcare units. The rapid opening and closing of units/wards, as part of this dynamic shift between crisis and normal management, contributed to increased workloads, including the reorganization of materials and routines. Anticipating the consequences of operational changes is crucial in managing resources effectively, especially during periods of constant adaptation [37, 40]. Improvements in the relocation process can benefit both transferred and permanent staff. Rather than daily adjustments, monthly relocations with standardized hospital-wide routines would help. Permanent staff endured most of the onboarding responsibilities for new team members while also caring for severely ill patients [14, 17, 35].

To optimize pandemic response, a two-step introduction process is recommended: one general orientation covering COVID-19-specific protocols managed by the HR department, and another specific to local unit practices. This structure prevents repetitive introductions and enables more efficient use of healthcare professionals [25, 36, 39, 43]. The suspension of elective healthcare services to prioritize care for COVID-19 patients created long-term challenges, including extended waiting lists. Therefore, managerial leadership is essential in maintaining a healthy work environment [11, 24, 37, 40]. Leadership skills, availability, and organizational commitment are key to supporting job satisfaction, teamwork, and collaborative healthcare practices [10, 36, 38–40, 44].

Despite improved knowledge of SARS-CoV-2 and evolving routines, the use of protective equipment posed ongoing challenges, heat, skin abrasions, headaches, and communication difficulties. Staff in “dirty zones” had to perform all care tasks independently. Structured schedules and standardization of equipment brands and procedures helped address these issues, improving continuity of care and patient safety [11, 23, 26, 37]. The study further emphasizes the significance of teamwork. Traditional “walls” between departments and professional silos dissolved, allowing collaborative, interdisciplinary efforts [45, 46]. The lack of expertise created a shared necessity for action without bureaucratic constraints. Staff adopted the *plan-do-study-act* method as a primary approach to ensure patient safety, eschewing hierarchy and enabling continuous improvement [43, 44].

This collaborative environment aligns with the principles of person-centred care [45, 46]. The focus on shared responsibility fostered a sense of belonging and unity rather than division between “them” and “us.” Together, healthcare professionals manage the pandemic through cooperation, not bureaucracy, doing what they do best: caring and curing.

### Limitations

This study was conducted at a single university hospital in Sweden and has both strengths and limitations. A notable strength is the inclusion of 28 participants from diverse professional and administrative backgrounds, providing a broad range of experiences in terms of education, age, and employment [25, 30]. One limitation, however, is that the participants were, on average, highly experienced (median age 49 years; median 21 years of professional experience). Therefore, future studies are recommended to explore the perspectives of younger staff with less experience, whose views on COVID-19 may differ.

All interviews were conducted by a trained researcher with no prior relationship to the participants, which minimized bias and enhanced the credibility of the findings. Nevertheless, the use of a single researcher may also be regarded as a limitation, as it could influence the quality of data analysis and the overall credibility of the study. To address this, the methodological description follows a rigorous scientific approach and is supported by relevant references to ensure soundness. The use of semi-structured interviews with follow-up questions further allowed participants to provide rich and detailed narratives. Data analysis was conducted using a well-established approach, qualitative content analysis [23, 24], with credibility reinforced through clear examples (Table 1) and direct quotations. Ethical principles were strictly observed [26–28], including written informed consent, voluntary participation, and anonymization of data to protect confidentiality. In addition, international references were incorporated in the conclusion to enhance the credibility of the study. Nonetheless, the study’s scope, restricted to a single hospital and 28 participants, represents a limitation. Therefore, further research is needed to broaden and validate these findings [1, 25].

## Conclusion

The study highlights how the rapid onset of the COVID-19 pandemic profoundly affected healthcare working conditions. Staff collaboration, supported by a non-bureaucratic approach and continuous improvement through the plan–do–study–act model, proved essential. Organizational and leadership efforts should prioritize the implementation of both short- and long-term strategies, including standardized onboarding processes with a clear structure outlining *what, when, how*, and *why in accordance to the specific healthcare units’ mission and demands*. These processes should distinguish between general and unit-specific orientations, delivered in group settings, to help alleviate the burden on both permanent and redeployed staff. Despite high workloads, the staff successfully adapted to the crisis through shared goals and commitment to patient safety. In conclusion, organizational commitment, free from rigid hierarchical structures, can ease pressure on healthcare staff and strengthen collaboration, partnership, and person-centred care.

## Data Availability

All data generated or analyzed during this study are included within this published article. However, the underlying dataset is not publicly available due to confidentiality and privacy considerations.
